# Three-dimensional printing models improve understanding of spinal fracture—A randomized controlled study in China

**DOI:** 10.1038/srep11570

**Published:** 2015-06-23

**Authors:** Zhenzhu Li, Zefu Li, Ruiyu Xu, Meng Li, Jianmin Li, Yongliang Liu, Dehua Sui, Wensheng Zhang, Zheng Chen

**Affiliations:** 1 Department of Neurosurgery, The Affiliated Hospital, Binzhou Medical University, Binzhou, Shandong, China; 2 Department of Endocrinology, The Affiliated Hospital, Binzhou Medical University, Binzhou, Shandong, China

## Abstract

Three-dimensional printing (3Dp) is being increasingly used in medical education. Although the use of such lifelike models is beneficial, well-powered, randomized studies supporting this statement are scarce. Two spinal fracture simulation models were generated by 3Dp. Altogether, 120 medical students (54.2% females) were randomized into three teaching module groups [two-dimensional computed tomography images (CT), 3D, or 3Dp] and asked to answer 10 key anatomical and 4 evaluative questions. Students in the 3Dp or 3D group performed significantly better than those in the CT group, although males in the 3D group scored higher than females. Students in the 3Dp group were the first to answer all questions, and there were no sex-related differences. Pleasure, assistance, effect, and confidence were more predominant in students in the 3Dp group than in those in the 3D and CT groups. This randomized study revealed that the 3Dp model markedly improved the identification of complex spinal fracture anatomy by medical students and was equally appreciated and comprehended by both sexes. Therefore, the lifelike fracture model made by 3Dp technology should be used as a means of premedical education.

Learning and identification of anatomy are a fundamental component for neurosurgery treatments. Practicing dissection of human vertebrae is the best method for improving the understanding of and surgery skills required for spinal fractures^1^,^2^. However, because of the scarcity of bones, not all medical students, even those staying in hospitals at all hours, can be provided this opportunity^3^. Therefore, we believe that if a model reflecting the condition could be used in class, numerous medical students could study and practice using this emulation bone model and benefit from it^4^,^5^.

3Dp is a fast and inexpensive technology of rapid prototyping (RP), a technology based on the construction of physical three-dimensional, layer by layer, according to their respective virtual models^6^,^7^. The model generated from scanning patients is sliced, and its transversal sections are physically reproduced through automated processes of layer-by-layer construction in powdered, solid, or liquid raw materials^1^,^2^,^6^. With the potential of spatial understanding, 3Dp is increasingly used not only in the clinical setting but also in the education of both medical students and patients^8^,^9^,^10^. However, in China, the clinical routine is almost exclusively characterized by two-dimensional (2D) images computed tomography (CT) and magnetic resonance imaging; some medical universities teach through 3D images^11^. Teaching combined with 3Dp technology is very rare, owing to the theory limitation.

We conducted a randomized controlled study for investigating the impact of 3Dp models on the identification of spinal fracture for medical students as well as for assessing sex-related differences in benefits compared with 2D and 3D presentations.

## Material and Methods

### Generating simulation fracture models by three-dimensional printing technology

Raw CT data of the spinal vertebrae from two patients were used: one had a fracture in the second cervical vertebra (C2), and another had a fractured ninth thoracic vertebra (T9). CT scans were taken in the horizontal plane using a slice width of 0.5 mm, a helical pitch of 2.5, and an image production interval of 0.1 mm. After scanning, data were exported to a DICOM file. Next, MIMICS 15.0 (Materialise, Belgium) was used for reconstructing a 3D image, which was converted to a **. STL (STereo Lithography) file from the DICOM file. Finally, the **. STL file was sent to the 3D printer (XYZ printing, China) based on fused deposition modeling (FDM) technology, whereas the sintered layers were accumulated using a helical pitch of 0.1 mm^12^. After assessment by two senior professors, no differences were found compared with the original fracture; therefore, the model was used in the teaching process as the next step.

### Medical education

**Figure 1:**
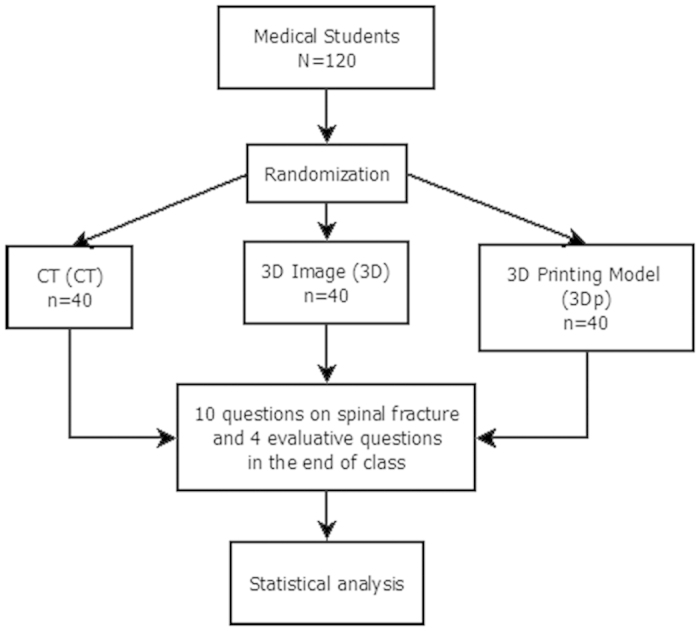
Flow chart of the study design.

All students completed their basic training by learning anatomy not only through textbooks and dissecting corpses but also through computer simulations in anatomy during their first or second year of enrollment in Binzhou Medical University. First, a lecture was given to the students, describing the spinal anatomy, and the definition of normal cervical and thoracic vertebra segments was presented using PowerPoint. Further, a short technical description of the teaching module (TM) was provided for helping the students in successfully completing the teaching process (Fig. 1).

**Figure 2:**
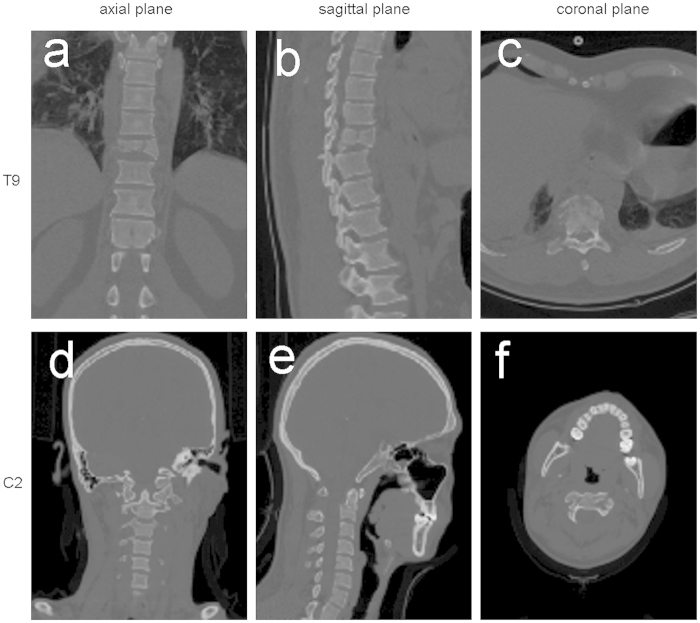
Screenshot of the teaching module for two-dimensional computed tomography images. The ninth thoracic vertebra (T9):**a, b**, and **c** (axial, sagittal, coronal views, respectively); the second cervical vertebra (C2): **d, e**, and **f** (axial, sagittal, coronal views, respectively).

**Figure 3:**
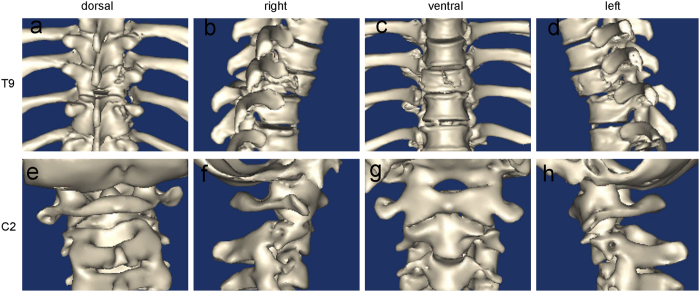
Screenshot of the teaching module for three-dimensional images. The ninth thoracic vertebra (T9): **a, b, c**, and **d** (dorsal, right, ventral, and left views, respectively); the second cervical vertebra C2: **e, f, g**, and **h** (dorsal, right, ventral, and left views, respectively).

**Figure 4:**
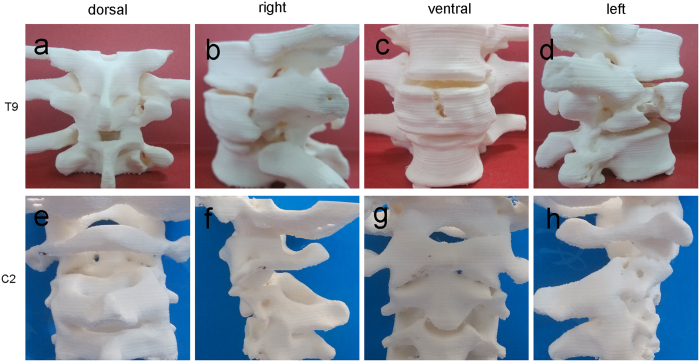
Photos of the teaching module for three-dimensional printing models. Two lifelike fracture models were reconstructed by a three-dimensional (3D) printer. The ninth thoracic vertebra (T9): **a, b, c**, and **d** (dorsal, right, ventral, and left views, respectively); the second cervical vertebra (C2): **e, f, g**, and **h** (dorsal, right, ventral, and left views, respectively)

Students were randomized into three groups using a computer program. The TM of the first group (CT group) was based on the CT images of the two patients in sagittal, coronal, and axial planes (Fig. 2). The TM of the second group (3D group) was based on the 3D images reconstructed from the DICOM file, original data file from the two patients, each angle freely adjustable in the computer (Fig. 3 and S1,2 Video). In the last group (3Dp group), each student received the two fracture models (Fig. 4 and S3,4 Video). A teacher provided a detailed interpretation of the fracture in each group.

Each group of 40 students was divided into seven subgroups in the teaching progress. Each subgroup was assigned six computers or 12 models. By the end of the class, each student answered 10 questions regarding spinal fractures and 4 evaluation questions (Table 1) about the TM, assessed by an examination paper, which contained many “3D images” (S Figure 1). In the examination paper, each student had to enter sex, school age in medical school, and a unique randomization code; an invigilator recorded the assignment time of each student. The answers to each question and the required time were recorded.

Students voluntarily participated through a hospital network post, and to protect their rights, no identifying information was recorded. This study was approved by the ethics review committee of Binzhou Medical College (20140108), and all students provided their written informed consent. All methods were performed in accordance with the approved guidelines.

### Statistical analysis

Statistical analysis was performed using SPSS (version 19.0 for Windows), and the level of statistical significance was set at the p value of <0.05. The distribution of continuous data was described using mean and standard deviation (SD). The data was analyzed using two-way analysis of variance (ANOVA) after testing for homogeneity. Tukey’s or Sidak’s multiple comparison test was used for post hoc analysis. In categorical data with absolute and relative frequencies (counts and percentages), potential differences between the groups were evaluated using chi-square tests. Box plots were used for visualizing the distribution of continuous data by GraphPad prism (version 6.0 for Windows).

## Results

### Fracture models were reconstructed by three-dimensional printing technology

Two lifelike fracture models were reconstructed by 3Dp technology, reflecting the situation of two patients with spinal fractures (Fig. 4 and S3,4 Video). Each model was constructed in 6 h at an approximate printing cost of US$ 20.

### Characteristics of students

In this randomized controlled study, a total of 120 medical students (54.2% females) participated, and no one dropped out. Forty students (22 females) were randomized into the CT group, 40 (21 females) into the 3D group, and 40 (22 females) into the 3Dp group, indicating no significant sex-related differences (Table 2). We also counted each student’s school age in medical university, and there was no statistically significant difference (Table 2).

### Sum scores of correct answers and time spent

**Figure 5:**
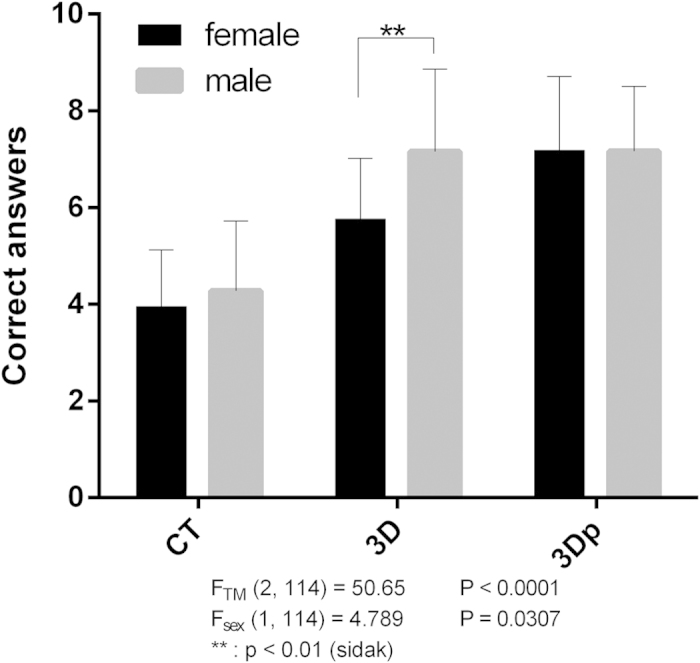
Sum score of correct answers. Students in the three-dimensional printing (3Dp) or 3D groups performed significantly better than those in the computed tomography (CT) group; males in the 3D group scored higher than females, in contrast to those in the 3Dp (post hoc by Sidak’s test) groups. F_TM_: F value of the three teaching modules; F_sex_: F value of sex; **p < 0.01.

We completed the randomized controlled study in three months. Overall, the results of the sum scores of correct answers (Table 3) were significantly different between the three groups affected by the TM and sex [F value of the three TMs (FTM) = 50.65, p < 0.0001 and that of sex (Fsex) = 4.789 p = 0.0307; two-way ANOVA]. Further, post hoc analysis revealed that students in the 3Dp and 3D groups performed better than those in the CT group based on Tukey’s multiple comparison test (3D vs. CT: mean difference (MD) = 2.325, p < 0.0001 and 3Dp vs. CT: MD = 3.075, p < 0.0001), while no significant differences were found between 3D and 3Dp groups (3D vs. CT: MD = 0.7500, p = 0.0508). Males in the 3D group scored higher than females (male vs. female: MD = 0.7500, p = 0.0508) by Sidak’s multiple comparison test; however, this phenomenon did not occur in the CT and 3Dp groups (Fig. 5).

**Figure 6:**
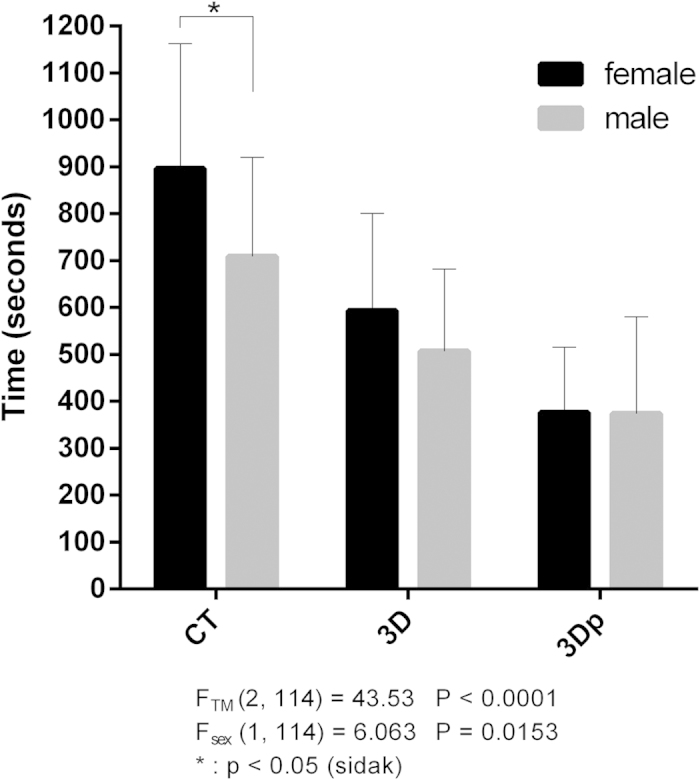
Overall time (s) required for answering the 10 questions. Students in the three-dimensional printing (3Dp) group were the first to answer all the questions (Tukey’s test), and there were no sex-related differences. However, more time was spent by females in the CT group. *p < 0.05.

Analysis of the time required for answering the 10 questions revealed a significant difference between imaging modalities: students in the CT group required 708.56 ± 212.08 s vs. 896.59 ± 266.08 s (mean ± SD s of male vs. female) for completing the 10 questions, whereas those in the 3D and 3Dp groups required 514.22 ± 177.69 s vs. 593.48 ± 207.36 s and 373.56 ± 206.97 s vs. 376.72 ± 138.95 s, respectively (Table 3). Post hoc analysis revealed that students in the 3Dp group were the first to answer all the questions (3Dp vs. CT: MD = −436.7, p < 0.0001; 3Dp vs. 3D: MD = −177.1, p = 0.0006; Tukey’s test), and there were no differences between males and females (female vs. male: MD = 3.172, p > 0.9999, Sidak’s test, Fig. 6).

### Answers to the evaluated questions

All students enjoyed the TM. However, students in the 3Dp (75%) and 3D (62.5%) groups answered the item “pleasure” (question #11) significantly more often with “yes, very much” compared with those in the CT group (37.5%; mean = 15.29, p = 0.004, chi-square test). The modality “assistance” (question #12) was rated “yes” significantly more often by students in the 3D (65%) and 3Dp (75%) groups compared with those in the CT (32.5%; mean = 18.02, p = 0.001, chi-square test) group. When interrogated about the personal learning effect (question #13), students in the CT group (47.5%) significantly more often answered “no,” whereas those in the 3D (60%) and 3Dp (70%) groups significantly more often answered “yes” (mean = 16.2, p = 0.003, chi-square test). Finally, significantly more students in the 3Dp (75%) and 3D (62.5%) groups felt that they could confidently explain the fracture to their fellow students (question #14) compared with those in the CT (25%; mean = 22.24, p = 0.000, chi-square test) group. Pleasure, assistance, effect, and confidence were predominant in students in the 3Dp group.

## Discussion

3Dp has been extensively used for designing and manufacturing prototypes in the fields of engineering and technology. In recent years, it has also been applied to medicine. There are five types of prototyping methods: those employing a stereolithography apparatus, selective laser sintering, and FDM and laminated object manufacturing and inkjet printing^13^. FDM is popular in hospitals and families in China because the involved printing process is cost-effective and has a high accuracy. In this randomized study, two authentic fracture models were reconstructed by this technology, reflecting the situation of spinal fractures (Fig. 4).

Altogether, 120 medical students (54.2% females) participated, and they were randomized into the CT, 3D, and 3Dp groups, with no significant sex- and age-related differences. Each student was asked to answer 10 key anatomical questions and 4 evaluative questions. Students in the 3Dp and 3D groups performed significantly better than those in the CT group, although males in the 3D, not 3Dp, group scored higher than females. Meanwhile, students in the 3Dp group were the first to answer all questions, and no sex-related differences were found. Students in the 3Dp group answered more positively than those in the CT and 3D groups to the 4 evaluation questions.

Sex-related differences were observed in understanding virtual images, which should not be ignored during teaching^14^,^15^. We found that males have an unfair advantage in understanding 3D image reconstructions compared with females. However, this sex-related difference was not present in the 3Dp group because teaching was based on real models.

3Dp is a “clone technology” that enables the generation of realistic models convenient for students to observe from any angle^12^,^16^,^17^,^18^. We also observed that using 3Dp objects improves the learning efficiency of medical students, helps them acquire expertise, and increases their interest and enthusiasm, which are important components in learning.

The application of 3Dp technology in medical education is rapidly developing. However, randomized controlled studies of medical education using 3D printed models remain scarce. Preece *et al*.^19^ developed and evaluated the use of a model demonstrating the complex spatial relationships of the equine foot, suggesting that physical models have a significant advantage over alternative learning resources in enhancing visuospatial and 3D understanding of complex anatomical architecture and that 3D computer models have significant limitations with regard to 3D learning. Moreover, many case studies regarding the benefits of 3Dp technology in teaching and training processes have been reported. Mashiko *et al*.^20^ developed a method for fabricating a 3D hollow and elastic aneurysm model for surgical simulation and training. They also presented a simulated 3D model of a human temporal bone made by the selective laser sintering method, based on detailed CT data, and it was found useful for understanding the 3D aneurysm structure. Cardiovascular anomalies have been presented in a study by Vranicar *et al*.^21^, where 3D models of blood vessels were analyzed for a better preoperative planning.

Despite our enthusiasm for this TM, our study has some limitations. First, there is a potential selection bias because the sample size was relatively small. Second, the master knowledge of each student may not have been entirely homogenous, which could have affected the stabilization, validation, and generalization of the final results. Third, the printing model and image data did not include analysis of muscles and neurovascular tissue surrounding the fracture, without which the model cannot completely represent a fracture condition. Finally, several technical problems, such as the lengthy printing time, needs to be resolved, which currently limits the applicability of the model.

Future advances in implementing 3Dp in medical education could include the development of printing devices that allow rapid onsite printing in the teaching hospital and development of 3Dp models that mimic the haptic characteristics of specific tissue (i.e., nerves, arteries, muscles).

## Conclusion

This randomized study revealed that the 3Dp model markedly improved the identification of complex spinal fracture anatomy by medical students and was equally appreciated and comprehended by both sexes. Therefore, the lifelike fracture model made by 3Dp technology should be used as a means of premedical education.

## Additional Information

**How to cite this article**: Li, Z. *et al*. Three-dimensional printing models improve understanding of spinal fracture—A randomized controlled study in China. *Sci. Rep.* **5**, 11570; doi: 10.1038/srep11570 (2015).

## Supplementary Material

Supplementary Information

Supplementary Video S1

Supplementary Video S2

Supplementary Video S3

Supplementary Video S4

## Figures and Tables

**Table 1:** Setting, question, and answers to the 14 questions.

**Table 2:** Characteristics of including students.

**Table 3:** Sum scores of correct answers and time spent.
