# Supplementary material for: Three-dimensional printing models improve understanding of spinal fracture—A randomized controlled study in China
Source: Sci Rep. 2015 Jun 23;5:11570. doi: 10.1038/srep11570 (PMC4477328; doi:10.1038/srep11570)
Supplement: Supplementary Information [file srep11570-s1.pdf]

Three-dimensional printing models improve understanding of spinal fracture—A randomized controlled study in China

Zhenzhu Li[&1], Zefu Li[&1*], Ruiyu Xu[2], Meng Li[1], Jianmin Li[1], Yongliang Liu[1], Dehua Sui[1], Wensheng Zhang[1], Zheng Chen[1].

[1] Department of Neurosurgery, The Affiliated Hospital, Binzhou Medical University, Binzhou, Shandong, China.

[2] Department of Endocrinology, The Affiliated Hospital, Binzhou Medical University, Binzhou, Shandong, China.

[&] Contributed equally.

Correspondence to lizefu@bzmc.edu.cn

Conceived and designed the experiments: LZF LZZ. Performed the experiments: XRY LZF LM ZWS CZ. Analyzed the data: XRY LZZ LJM LYL SDH. Wrote the paper: LZF LZZ. All authors reviewed the manuscript.

Competing Interests: The authors have declared that no competing interests exist.

Supporting information

S Figure 1. Examination paper in English

S1 Video. Three-dimensional view recorded on the computer screen of the ninth thoracic vertebra

S2 Video. Three-dimensional view recorded on the computer screen of the second

cervical vertebra

S3 Video. Three-dimensional printing model video of the ninth thoracic vertebra

S4 Video. Three-dimensional printing model video of the second cervical vertebra

S Figure 1. Examination paper in English as follows:

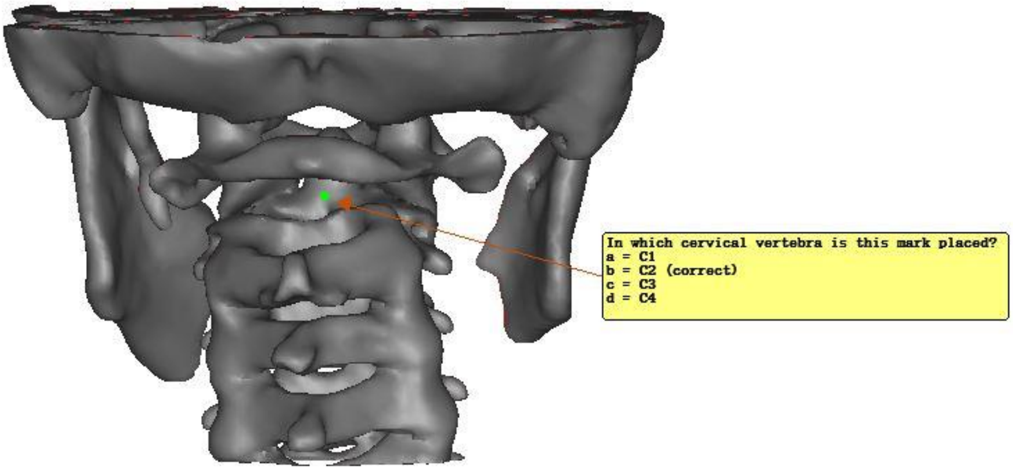

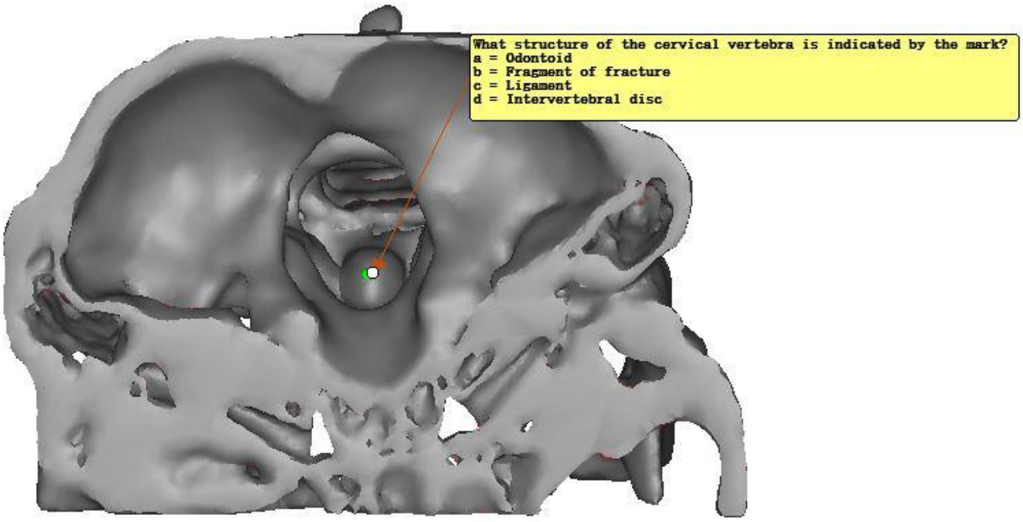

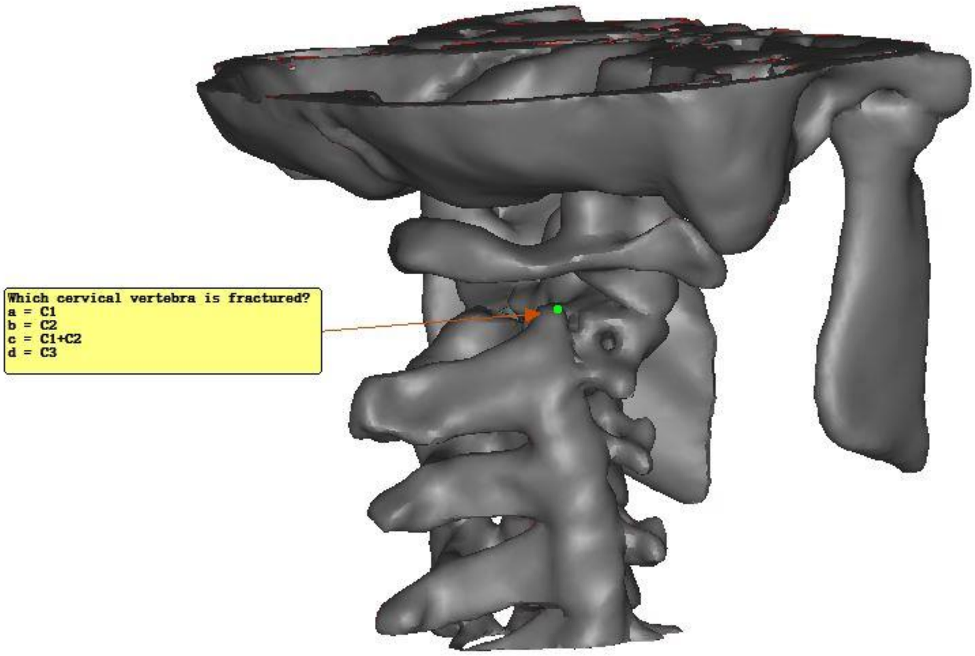
Which cervical vertebra is fractured?
a = C1
b = C2
c = C1+C2
d = C3

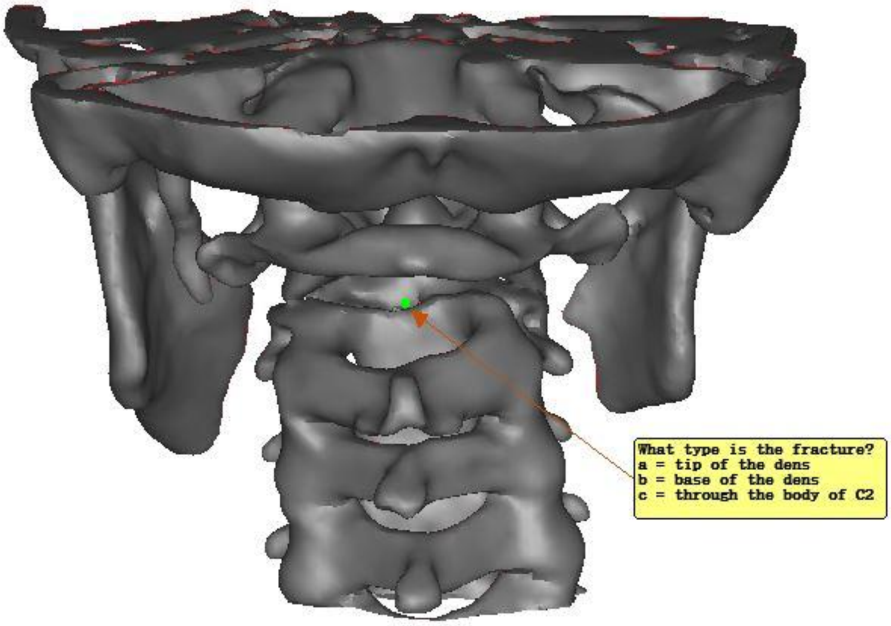
What type is the fracture?
a = tip of the dens
b = base of the dens
c = through the body of C2

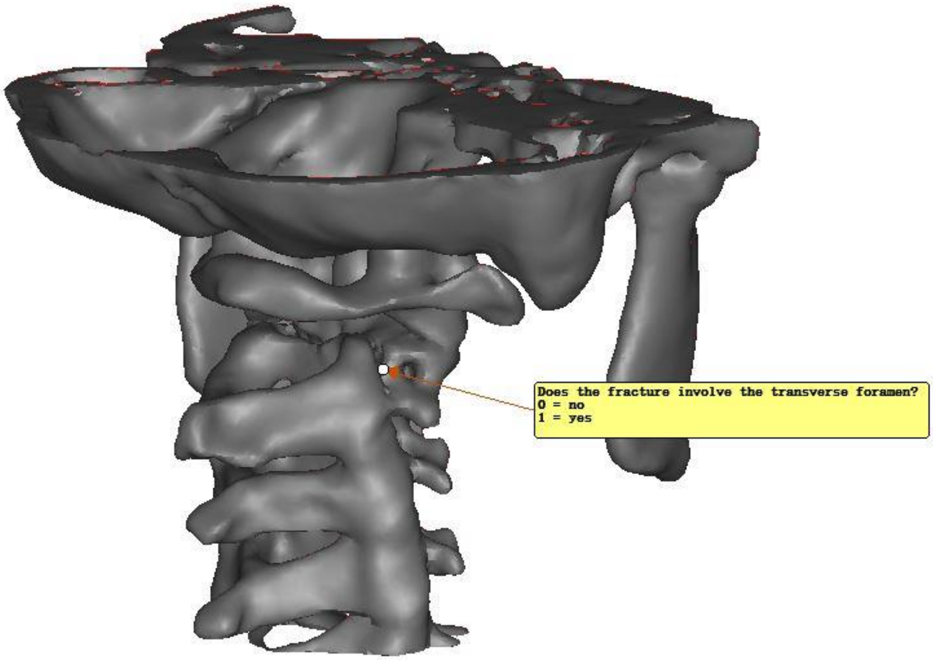
Does the fracture involve the transverse foramen?
0 = no
1 = yes

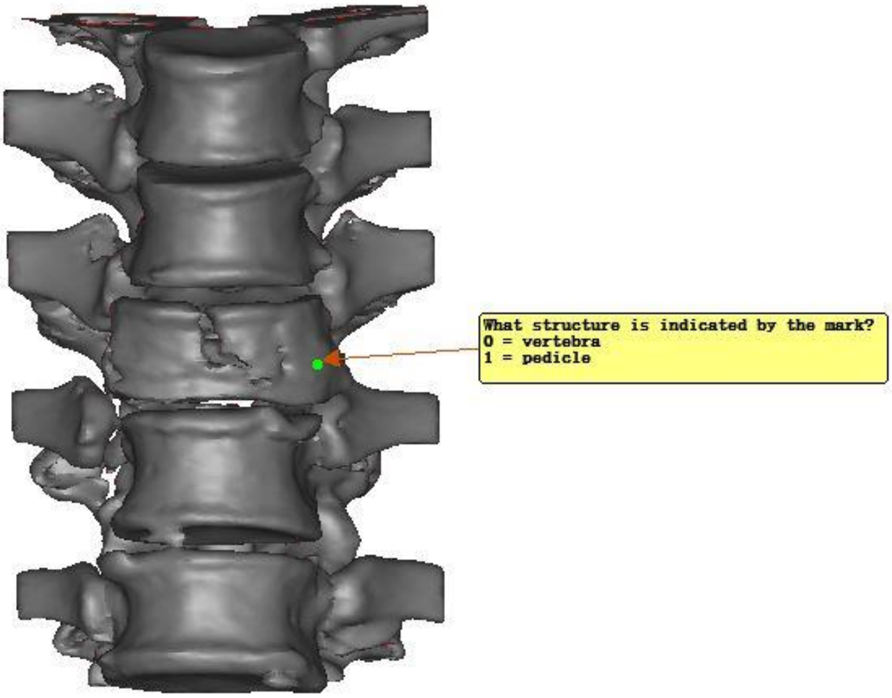
What structure is indicated by the mark?
0 = vertebra
1 = pedicle

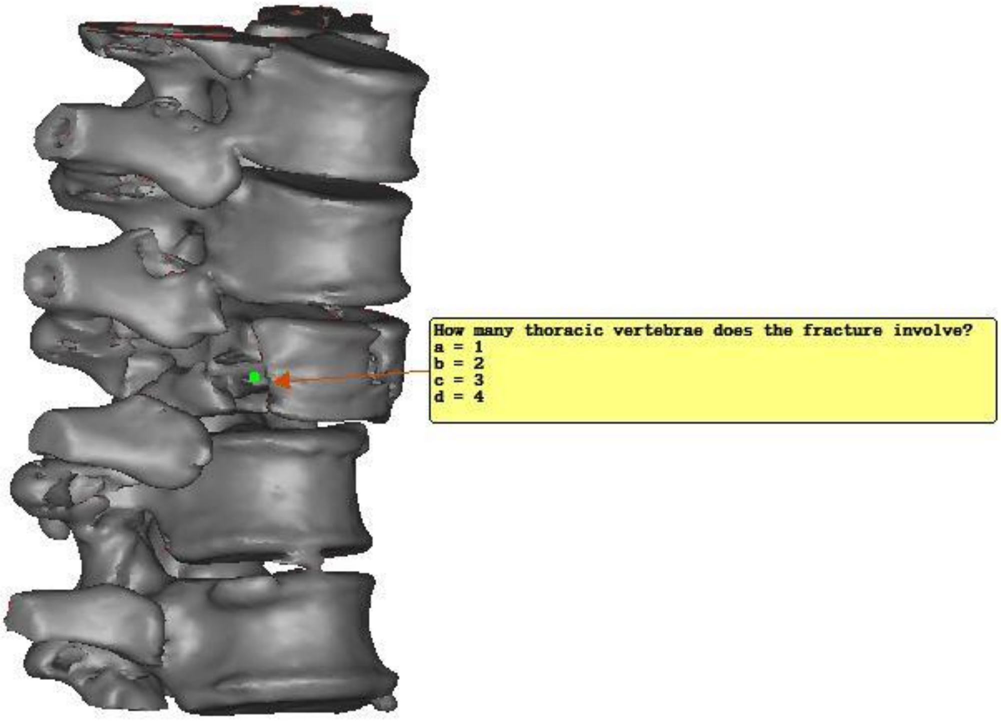
How many thoracic vertebrae does the fracture involve?
a = 1
b = 2
c = 3
d = 4

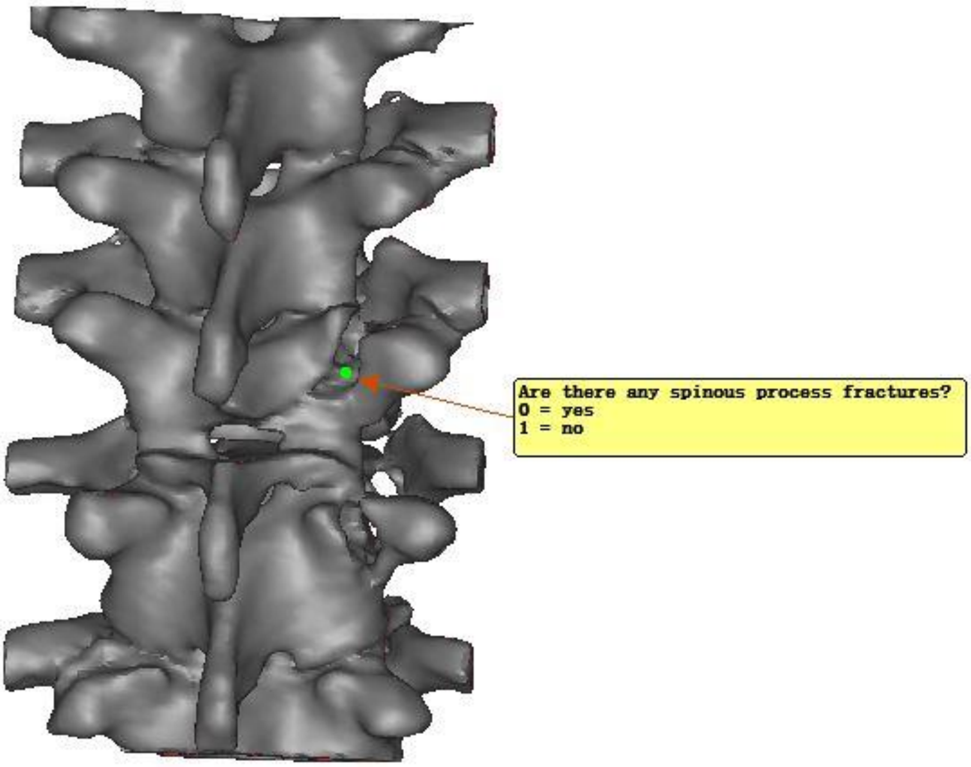
Are there any spinous process fractures?
0 = yes
1 = no

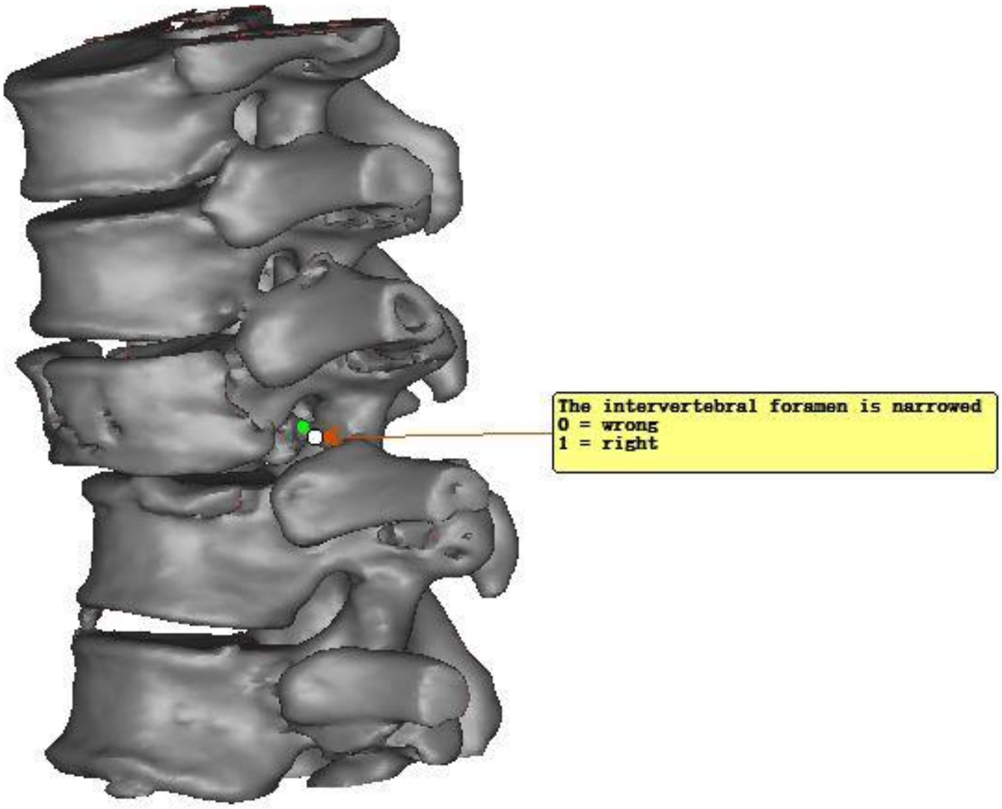
The intervertebral foramen is narrowed
0 = wrong
1 = right

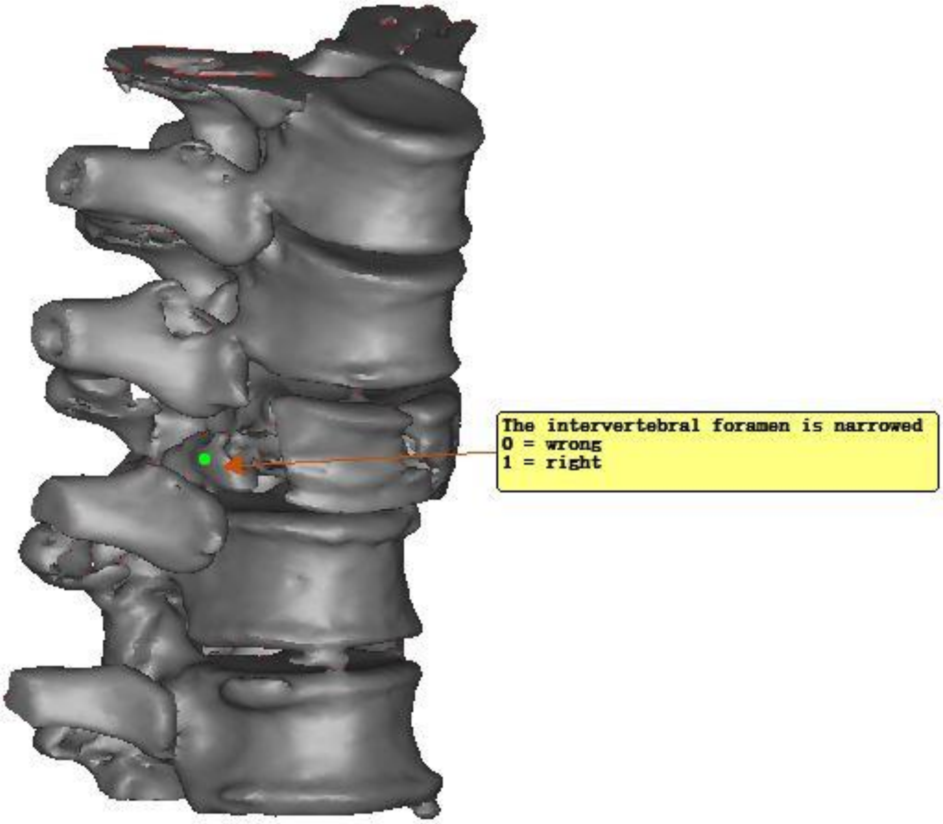
The intervertebral foramen is narrowed
0 = wrong
1 = right
